# Simultaneous Multiparameter Cellular Energy Metabolism Profiling of Small Populations of Cells

**DOI:** 10.1038/s41598-018-22599-w

**Published:** 2018-03-12

**Authors:** Laimonas Kelbauskas, Shashaanka P. Ashili, Kristen B. Lee, Haixin Zhu, Yanqing Tian, Deirdre R. Meldrum

**Affiliations:** Center for Biosignatures Discovery Automation, The Biodesign Institute, Arizona State University, 1001 S. McAllister Ave., Tempe, AZ 85287 USA

## Abstract

Functional and genomic heterogeneity of individual cells are central players in a broad spectrum of normal and disease states. Our knowledge about the role of cellular heterogeneity in tissue and organism function remains limited due to analytical challenges one encounters when performing single cell studies in the context of cell-cell interactions. Information based on bulk samples represents ensemble averages over populations of cells, while data generated from isolated single cells do not account for intercellular interactions. We describe a new technology and demonstrate two important advantages over existing technologies: first, it enables multiparameter energy metabolism profiling of small cell populations (<100 cells)—a sample size that is at least an order of magnitude smaller than other, commercially available technologies; second, it can perform simultaneous real-time measurements of oxygen consumption rate (OCR), extracellular acidification rate (ECAR), and mitochondrial membrane potential (MMP)—a capability not offered by any other commercially available technology. Our results revealed substantial diversity in response kinetics of the three analytes in dysplastic human epithelial esophageal cells and suggest the existence of varying cellular energy metabolism profiles and their kinetics among small populations of cells. The technology represents a powerful analytical tool for multiparameter studies of cellular function.

## Introduction

Cellular communication plays a central role in tissue homeostasis and disease states. Cancer is viewed as cells evading normal cell functionality through complex alterations in their signalling cascades and through cellular communication within the microenvironment^1^. Most of the current analytical approaches used to understand cancer and other diseases are based on performing assays with large populations of cells (>10^4^). The information obtained with these approaches represents an ensemble average of the response from the entire cell population, while completely obscuring the details about a possible spectrum of responses due to the presence of aberrant sub-populations of cells or even individual cells^2^. Elucidating such heterogeneous information about the composition of cell populations has the potential to reveal a detailed view of the disease state in the context of multicellular complexity by providing deep insight into cellular function. Cellular communication can occur in various ways and its role has been demonstrated in a variety of diseases. Mutations in genes encoding proteins of gap junction channels, one form of cellular communication, have been associated with deafness^3^, and sudden infant death syndrome^4^, while also being identified as a therapeutic target for reducing the spread of traumatic brain injury^5^ and heart injury^6^. Cell-to-cell communication via tight junctions has been shown to play an important role in cell proliferation^7^ and differentiation^7,8^, and has been implicated in a variety of diseases including cancer^9–13^. Cellular communication in cancer plays a key role in the tumor microenvironment facilitating tumor growth and metastasis^14,15^. The notion of cell-to-cell communication has also been reinforced by the finding that clusters of circulating tumor cells (CTCs)^16–19^ exhibit a significantly higher metastatic potential as compared to single CTCs^20^. This indicates that cell-cell interactions play a central role in metastasis formation and development.

Developing technological platforms addressing the need of analysing the heterogeneity of cellular function in the presence of cell-to-cell communication represents a formidable challenge. One faces the difficulty of dissecting the responses of individual cells or small populations of cells in a larger, heterogeneous population of cells with overlapping responses. On the other hand, while single-cell analysis approaches that are based on monitoring cellular function in individual cells in isolation address directly cellular heterogeneity^21–25^, their main disadvantage is the absence of cellular communication. As a compromise between the analysis of large populations of cells and individual cells, it is conceivable that one can utilize populations of communicating cells that are small enough to alleviate the ensemble averaging effect over thousands of cells with varying responses. Such a modality requires the generation of cell populations containing small, on the order of few to tens of cells, and controllable numbers of cells situated in close proximity, and an analysis system with adequate sensitivity and specificity to detect the relatively weak signals from such small numbers of cells.

Cell patterning using various cell-adhesive proteins, such as laminin for pancreatic β cells^26^, fibronectin for mammalian^27^, and endothelial cells^28^ has been reported. In one such study^29^ the authors explored the generation of spots of extracellular matrix (ECM) with two different dimensions: 20 × 20 µm, and 40 × 40 µm, for cell localization. It has been shown that the average number of cells per spot for the 20 × 20 µm and 40 × 40 µm geometries was 1.3, and 3.1, respectively. Our group has recently reported a non-contact method for the generation of small (<100 cells/population) populations of epithelial cells with high consistency^30^. Microcontact printing for developing arrays of cell adhesion regions, and cell adhesion protein for cell adhesion, are the methods of choice to achieve such cell cluster patterning. In this paper, we use cell adhesion promoting proteins for selective adhesion of cells, but without microcontact printing, thus reducing the number of fabrication steps and simplifying the technique.

Commercial bio-analytical instruments that are used to explore cellular energetics, such as the Seahorse XFp analyzer (Agilent Technologies, Santa Clara, CA)^31^, and Oxygraph 2k (OROBOROS Instruments, Innsbruck, Austria)^32^ require a at least 10^4^ cells per assay. It is important to note that the capability of these instruments is largely limited to extracellular sensing and the ability to analyze biological samples of limited size (for example, circulating tumor cells (CTCs)) has not been demonstrated. Technological advances tailored towards functional analysis of individual cells such as: cell manipulation^33,34^, detection of analytes^35–38^, isolation^39^, and bioanalytical instrumentation^22–24^ offer potent tools for the analysis of small populations of cells. A microchamber-based device for measuring the oxygen consumption rate (OCR) of single and multiple cells^21,22,36–38^ with a sub-fmoles/min resolution, combined sensing of OCR and extracellular acidification rate (ECAR)^24^, and combined sensing of OCR and measuring gene expression levels of the same cells^23^ have been reported. These single and multiple parameter measurements have demonstrated the importance of single-cell analysis by showing substantial variations in OCR and ECAR within isogenic populations of cells. The dimensions of the wells used to perform single cell analysis, however, limit the number of cells that can be placed in a single well. In addition to the limitations imposed by the well dimensions, the deposition of a larger number of cells into wells using the cell isolation system reported in^33^ is technically challenging.

Building on the above mentioned technologies of cell adhesion proteins^26–28,30^ and microchamber-based design^21–24^, we report a new technology that allows simultaneous quantification of multiple extracellular and intracellular physiological parameters of small (<100 cells) populations of cells. The technology is based on the creation of an isolated microenvironment for the cell population, for measuring transmembrane fluxes of rapidly diffusing small molecules involved in the bioenergetic machinery such as oxygen and protons. As a result, we report simultaneous measurements of OCR, ECAR, and mitochondrial membrane potential (MMP). This report represents a proof-of-principle of the technology, and demonstrates its capability to perform multiparameter functional analysis of small populations of cells. The technology can be used with a combination of different types of commercially available intracellular fluorescence sensors, including fluorescent protein reporters, and can be readily expanded to accommodate larger numbers of extracellular sensors, such as reported in^36–38,40–49^ to include other analytes of interest for real-time characterization of the cellular function. The scalable microchamber design is compatible with next generation microfluidic devices for metabolic profiling of cells in response to stimulus at the single-cell, multiple-cell, and tissue-level.

## Results and Discussion

### Microchamber validation

**Figure 1:**
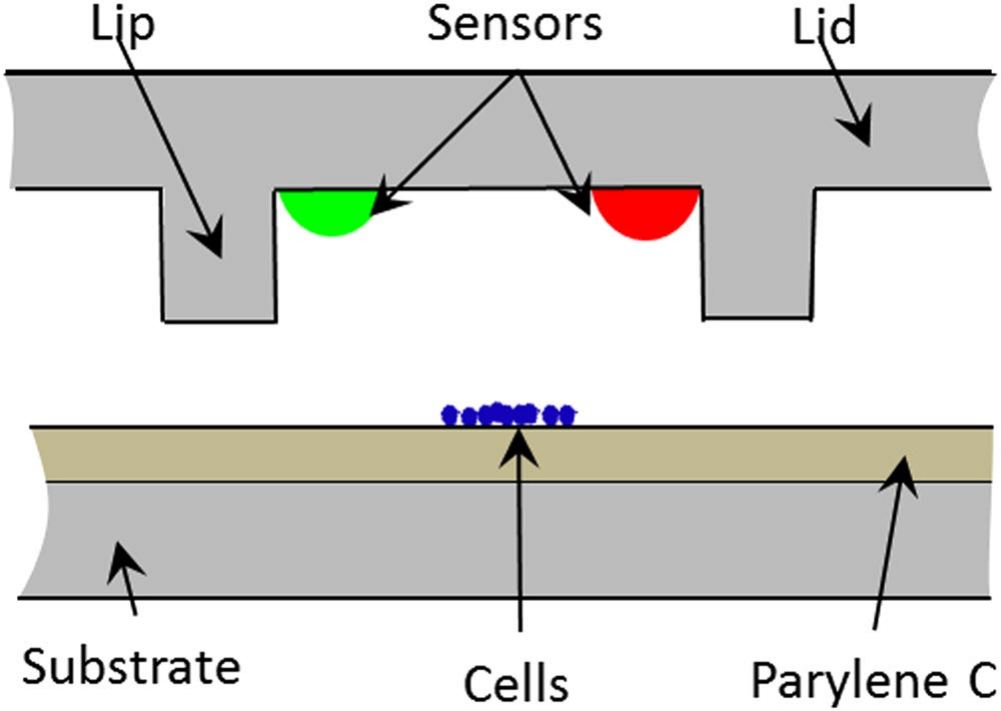
A cross-sectional view of the microchamber with multiple extra-cellular sensors in the lid and a cell colony on Parylene C coated substrate. Both the lid and the bottom substrate are microfabricated in glass (fused silica) using wet-etch lithography. The lipped lid is used to facilitate hermetic sealing between the two lid and bottom substrate of the device. Once assembled, the two parts form a hermetically sealed chamber containing the cell colony. The amounts of analytes of interest inside the chamber can then be measured in real time by analyzing the changes in sensor intensities. A 3 × 3 array of such microwells was used in this study.

**Figure 2:**
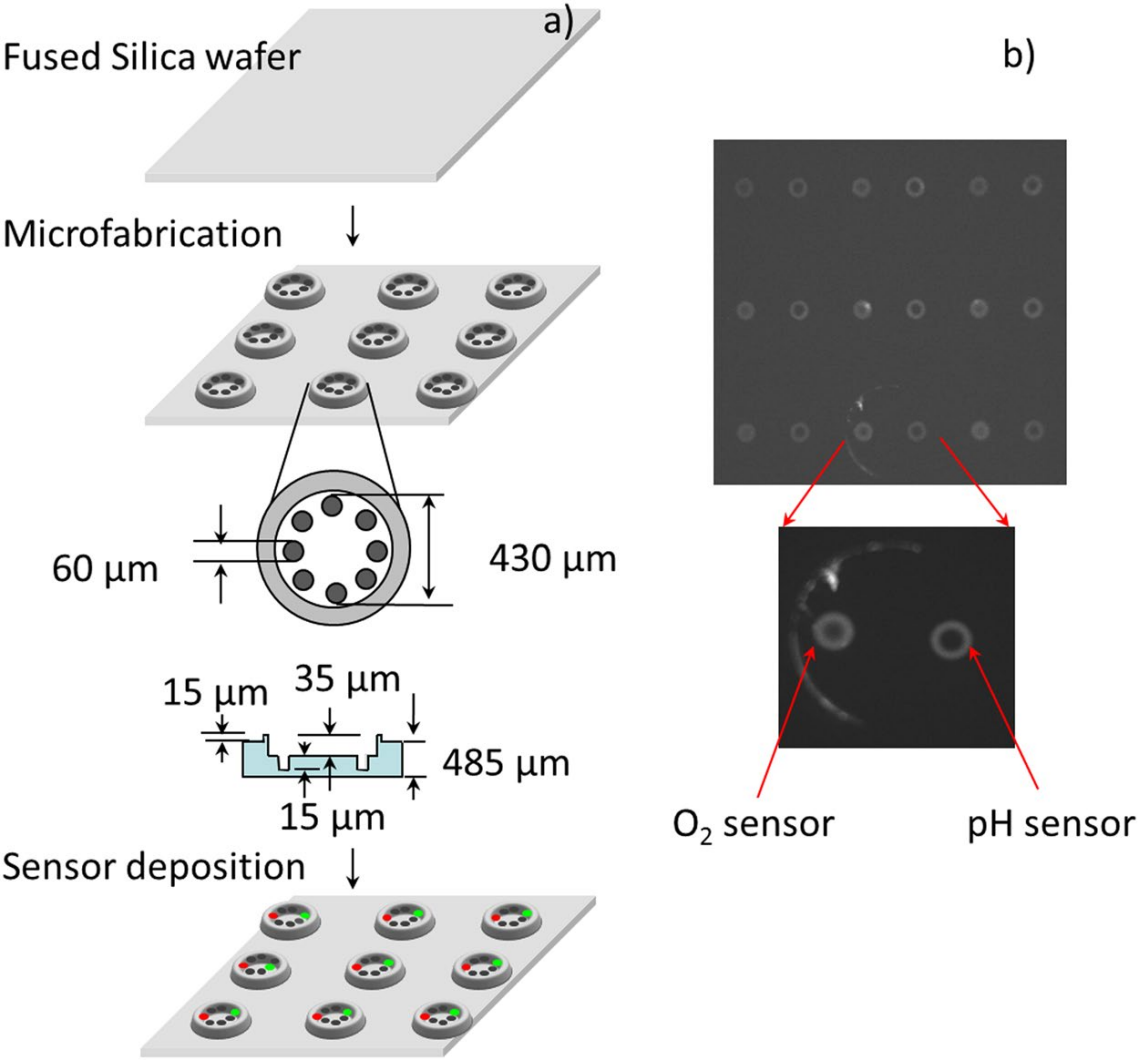
Lid with micro-pockets design for simultaneous monitoring of up to 8 different analytes. (**a**) Process flow and lid dimensions (not to scale). (**b**) Fluorescence emission micrograph of a 3 × 3 array of micro-pocket lids containing the oxygen and pH sensors.

**Figure 3:**
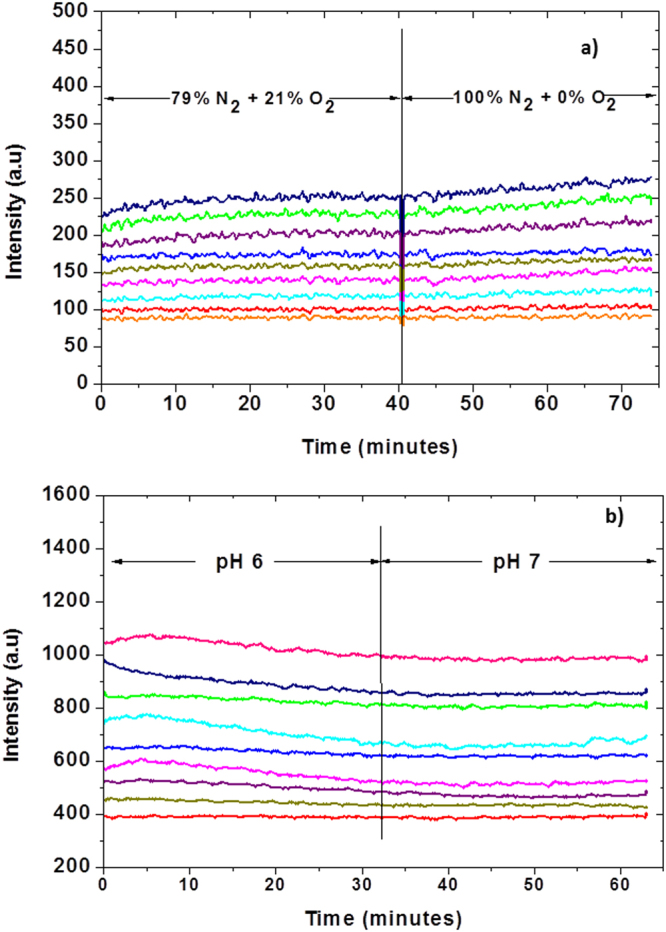
Hermetic sealing test of the device. The hermetic seal of the assembled microchambers was validated for oxygen (**a**) and proton (H^+^, (**b**)) leak by purging outside media with a mixture of gas containing different concentrations of oxygen and nitrogen or using buffers with different pH values, respectively. The lack of change in emission intensity of each of the corresponding sensors indicates hermetically sealed microchambers. We note that the minor (about 10%) increase in the oxygen sensor intensity in some of the microchambers over a time period of 35 minutes after oxygen concentration in the gas mixture was reduced to zero is negligible and can be corrected, as the sensor response during an assay is typically 100–150% in comparable period.

The ability to produce hermetically sealed microchambers (Fig. 1) was tested for each lid prior to assays. To this end, the microwell array containing the sensor (“lid”, Fig. 2) attached to the bottom of a piston mounted on a motorized XYZ stage was placed on top of a substrate coated with Parylene C, with a 2.7 kg weight on top of the piston forming a closed “microchamber”. Once the microchambers were formed, the air-saturated cell culture media around the microchambers was purged with a mixture of gas containing different known concentrations of oxygen and nitrogen. While purging, the fluorescence intensity of the oxygen sensor in the lids was monitored as a function of time, over a period of 60–80 minutes which is comparable to the typical time of an assay. Figure 3 shows the results of intensity plots of the oxygen and pH sensors. The oxygen sensor shows no or negligible (<20%), intensity change in response to the change in outside oxygen concentration (Fig. 3A). Similarly, negligible intensity response of the pH sensor can be observed in response to the change in the outside pH (Fig. 3b). Both tests indicate that the microchambers are hermetically sealed for both oxygen and pH.

### Sensor calibration

**Figure 4:**
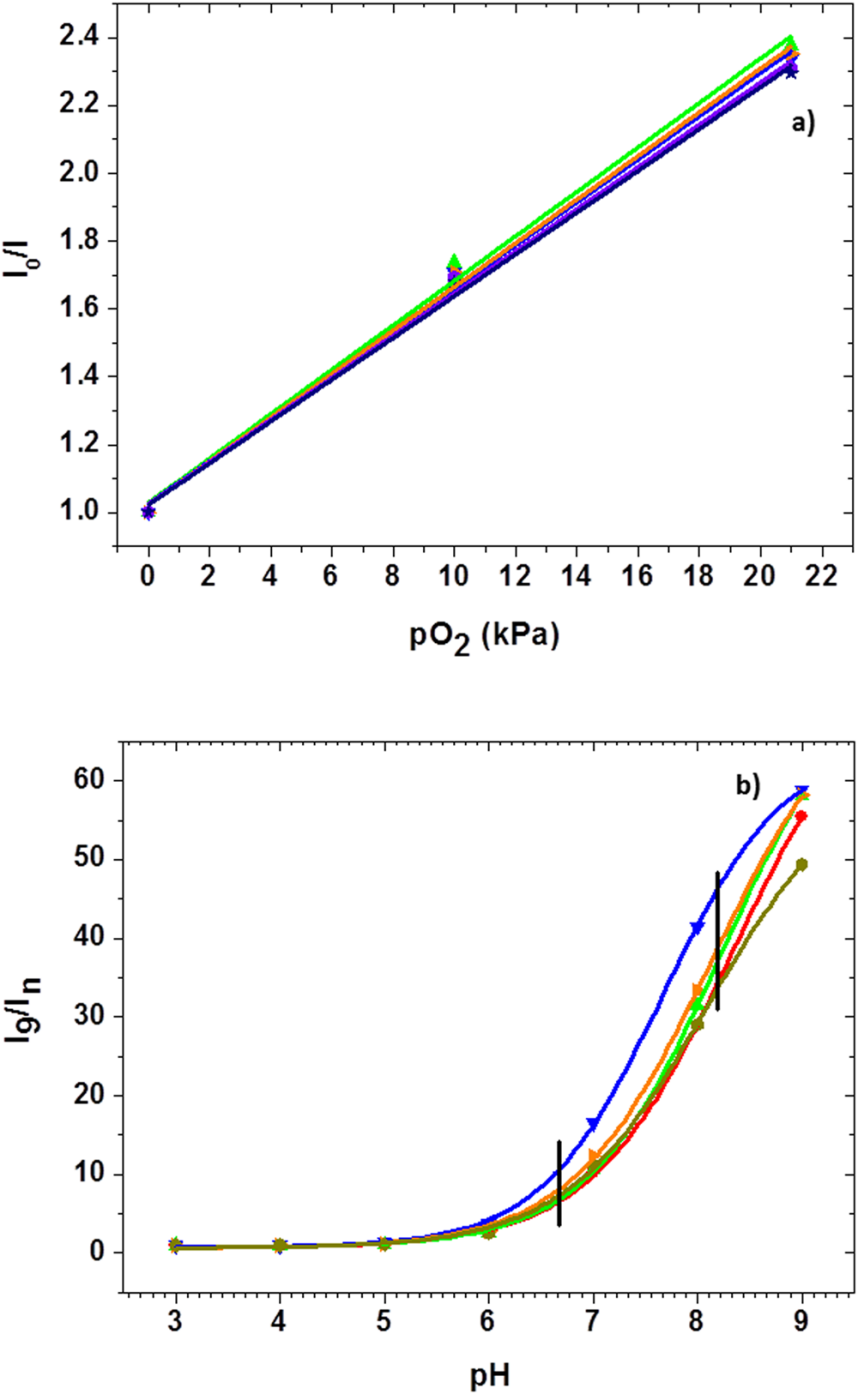
Calibration of the oxygen and pH sensors in five different micro-pockets on the same lid. (**a**) Stern-Volmer graph for oxygen sensor calibration shows a linear response to increasing oxygen concentration. The oxygen sensor was calibrated in DMEM medium purged with a gas mixture containing varying oxygen concentration. (**b**) pH sensor calibration with Boltzmann curve fitting. The sensor response in a pH range of 6.7–8.2 (black vertical lines) can be approximated as linear. The calibration was performed in a series of Britton-Robinson buffers with differing pH values.

The O_2_ and pH sensors were calibrated by varying corresponding variables over time. For oxygen sensor calibration, the lid containing sensor was immersed in cell culture medium, and different dissolved O_2_ concentrations were achieved by purging the solution with mixtures of N_2_ and O_2_ gas of known concentrations. The mixing of gas was done in a mass flow gas manifold controller (MC10SLMP-D, Alicat Scientific, Tucson, AZ). For pH calibration, the lid was immersed in a Petri dish containing a 5–7 mL of Britton-Robinson buffer with known pH value. Buffers with differing pH were used to measure the sensor response. The experiment was performed in a pH range of 3–9. Figure 4 shows the calibration results of the oxygen (a) and pH (b) sensors. The Stern-Volmer graph in Fig. 4a shows the intensity response curves corresponding to different oxygen partial pressures for five different lids of a 3 × 3 array. The response curves have had a linear curve fit applied, using linear regression. The response of the pH sensor was fitted with a sigmoidal (Boltzmann) function (Fig. 4b**)** using the following equation:

1 $$\frac{{I}_{9}}{I}={A}_{2}+\{\frac{({A}_{1}-{A}_{2})}{1+{e}^{\frac{x-{x}_{0}}{dx}}}\}$$

where I_9_ is the intensity at the highest pH (pH = 9) value used in the measurements, I is the intensity at any given pH, A_1_ and A_2_ are the initial and final values, x_0_ is the point of inflection, dx is the width of the sigmoidal curve, and x is the corresponding pH value. The R^2^ value of the individual curves is greater than 0.9995, indicating a reliable fit. The average pKa of the nine sensors from a 3 × 3 array is 8.2 with a range of 7.7–8.3.

All sensor lids were calibrated and checked for the ability to create hermetic seal prior to use. Lids with sensors that deviated from the Stern-Volmer (oxygen) or sigmoidal (pH) behaviour or failed the hermetic seal test were discarded. The average yield of quality lids was about 50%. We note that a lid, once calibrated and checked for hermetic seal generation, can be used repeatedly given proper cleaning steps between the experiments to avoid cross-contamination.

### Physiological measurements

**Figure 5:**
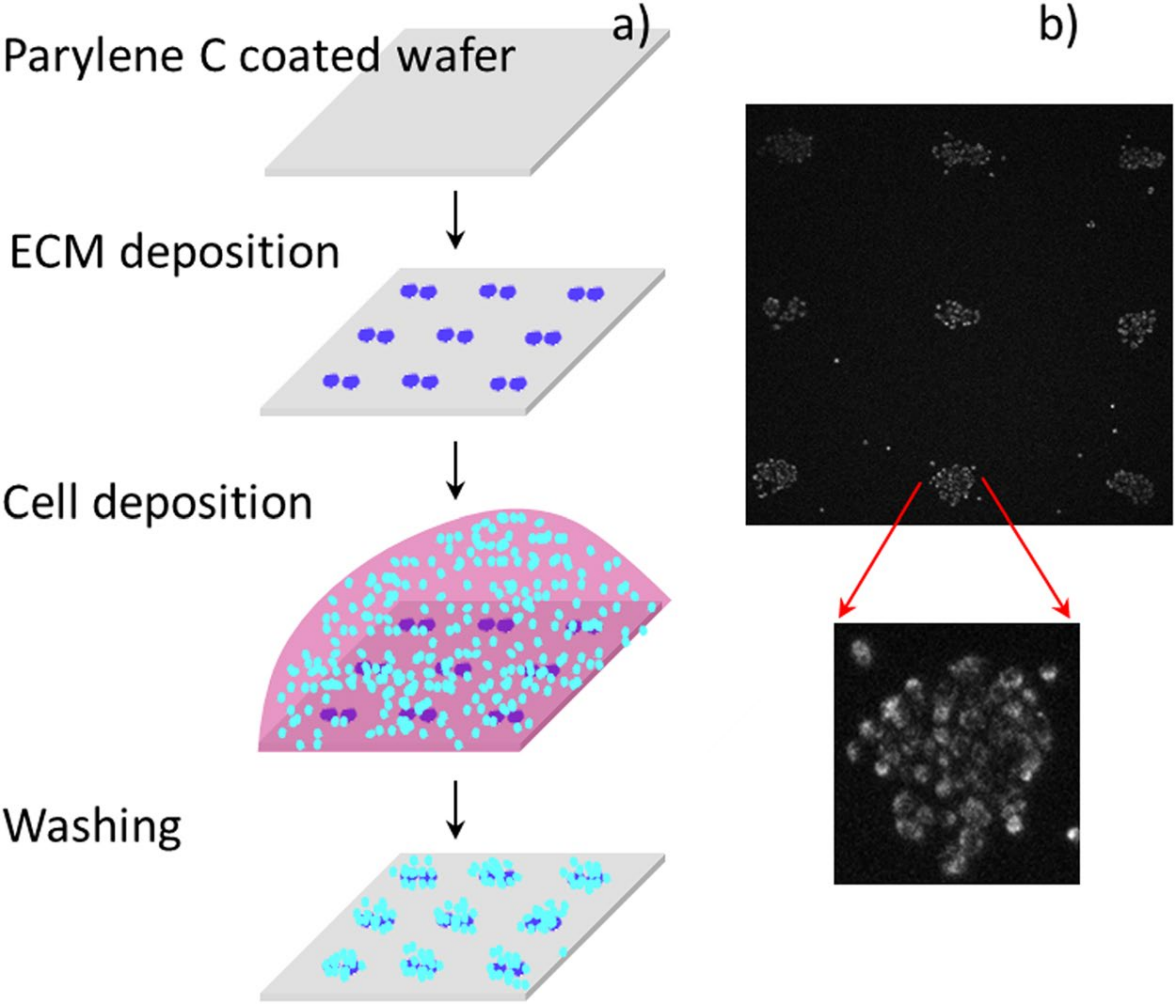
Formation of small cell colonies on the bottom substrate. (**a**) Process flow. First, to facilitate preferential adhesion to specific areas on the substrate, a solution of the extracellular matrix (ECM) protein fibronectin is deposited using a non-contact piezoelectric printing approach^30^; Second, cells suspended in culture medium are deposited and allowed to gravity-settle on the substrate; Third, the substrate is washed with PBS 3 times to remove the weakly adhered cells from the interstitial areas. (**b**) Fluorescence emission micrograph of cell colonies stained with JC-1 after 2 hours of incubation following deposition. The cell colonies are created in a 3 × 3 pattern matching that of the microwells on the top (lid) substrate of the device. A zoom-in on one of the colonies shows compactly positioned cells.

**Figure 6:**
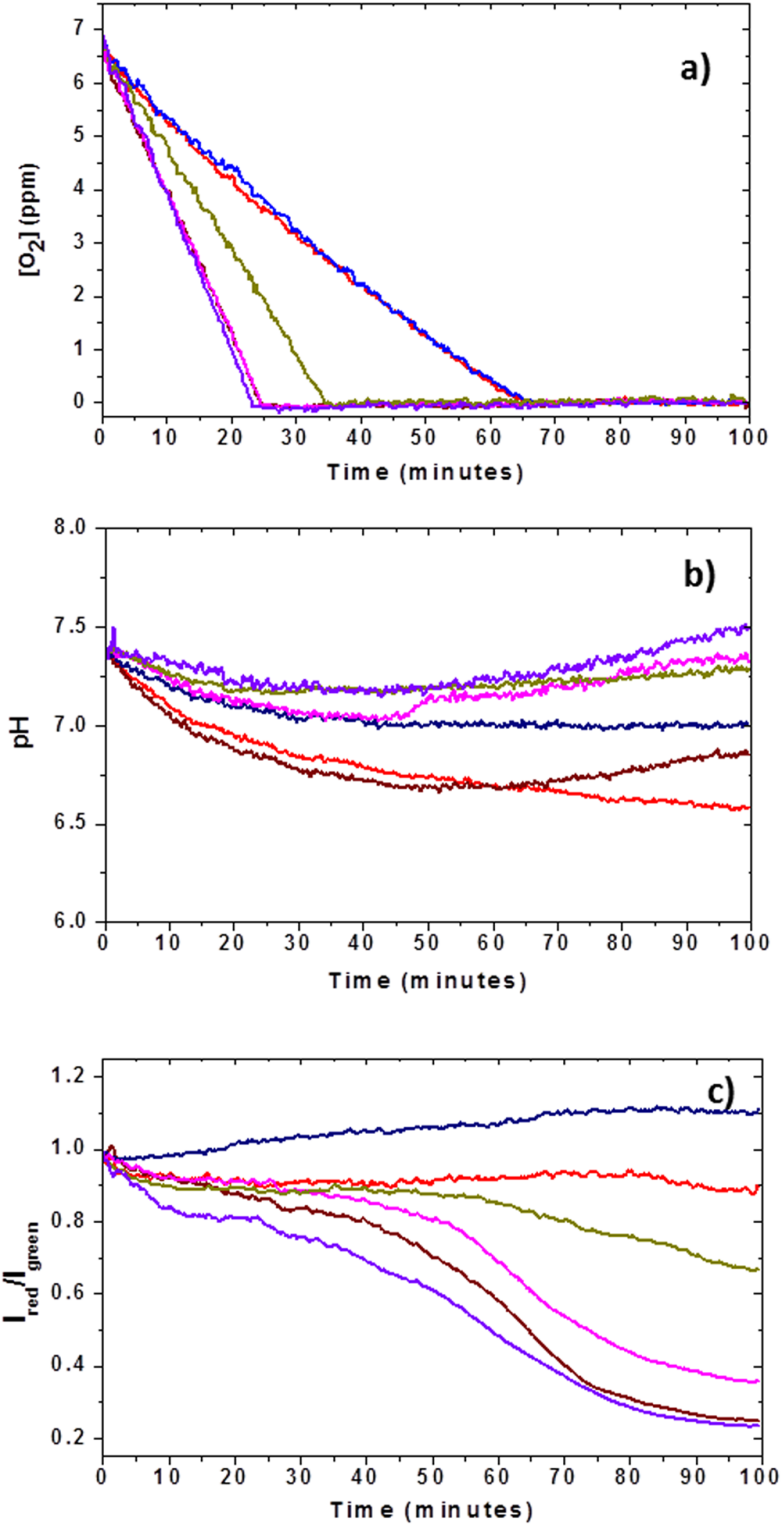
Simultaneous multiparameter physiological measurements of (**a**) oxygen consumption, (**b**) extra-cellular acidification and (**c**) mitochondrial membrane potential (MMP). For clarity, the data obtained from the same cell population are color-coded with the same color across all three graphs. While oxygen consumption kinetics are predominantly linear until all oxygen is consumed (inflection point in the kinetics), pH and MMP kinetics are highly variable and non-linear. After an initial decrease, the pH kinetics of some of the populations (pink, purple, and brown curves) showed an increase in pH value, while the remaining populations exhibited decreasing pH for the entire duration of the assay. The MMP kinetics differed strongly among the populations, with some maintaining or even slightly increasing the MMP levels (red and blue curves, correspondingly). The population (cluster) sizes are as follows: red −51, blue −46, beige −40, purple −42, pink −38, brown −39 cells. Note: the brown curve in oxygen consumption kinetics (panel a) is invisible due to a strong overlap with the pink and purple curves.

Data has been acquired from a total of 123 clusters, with 87 clusters stained with Hoechst 33342, and 36 clusters stained with JC-1. The cell clusters were deposited on flat substrates patterned with a mixture of ECM proteins (Fig. 5). The data (Table 1) was normalized against the cell count in each cluster. The number of responding cell populations per experiment with a 3 × 3 array device varied from 1 to 9, with an average of 5 responding population per experiment. The data were collected from a total of 23 successful experiments, where “successful” means at least one of the 9 wells in the device provided valid readout. Representative real-time measurements of the kinetics of oxygen consumption, extracellular acidification, and mitochondrial membrane potential (MMP) of six cell populations placed on the same substrate are shown in Fig. 6. All data points were collected with an 8 second interval between two consecutive measurements (Materials and Methods). The O_2_ and pH sensor intensity data were converted into ppm and pH units, correspondingly, using the conversion factors calculated from the previously acquired calibration data (Materials and Methods). For direct comparison of the kinetics, each MMP curve was normalised to the corresponding intensity value at the time point 0. Occasionally, there was a spatial overlap between the cell population stained with JC-1 and the extracellular O_2_ and pH sensors in the lid, thereby leading to a spectral cross talk in detected fluorescence signal primarily between the pH sensor and the JC-1 monomers. Efforts were made to eliminate the spatial sensor overlap by manually re-aligning the lid. In cases where this was not possible, the data obtained from the cluster was discarded. Due to the complex (changing kinetics) temporal profile of the extracellular pH (Fig. 6b), only the initial 150 data points corresponding to the first 20 minutes of the assay were utilized in calculating extracellular acidification rate (ECAR). For oxygen consumption rate (OCR) calculations, all data points in the oxygen consumption kinetics curves were taken into account. Because the calibration of the pH sensor indicates a linear relationship between pH and intensity within a pH range of 6.75–8.3 (Fig. 4b), the intensity values of the pH sensor were converted to the pH values using the following linear equation:

2 $$pH=\frac{((\frac{{y}_{c}\,{I}_{n}}{{I}_{o}})-{C}_{c})}{{m}_{c}}$$

where I_n_ represents experimental intensity value for which corresponding pH value needs to be calculated, I_0_ represents the initial experimental intensity value, y_c_ represents the normalised value corresponding to pH of the initial medium (pH = 7.4), C_c_ represents the intercept value of the calibration curve, and m_c_ represents the slope of the calibration curve. All y_c_, C_c_ and m_c_ values are obtained by averaging the values of the nine sensors from a 3 × 3 array in each lid.

**Table 1:** Statistical description of OCR and ECAR.

CP-D (n = 123)	Mean	SD
OCR (fmoles·min^−1^·cell^−1^)	0.63	0.186
ECAR (mpH·min^−1^·cell^−1^)	0.5	0.26

The oxygen sensor response was modelled using the Stern-Volmer relationship between dissolved oxygen concentration and sensor emission intensity:

3 $$\frac{{I}_{0}}{I}=1+{k}_{SV}[{O}_{2}]$$

where I_0_ is the emission intensity at zero oxygen concentration, I is the measured emission intensity; k_SV_, is the Stern-Volmer quenching constant; and [O_2_] is dissolved oxygen concentration. The k_SV_ and I_0_ parameters were determined using a three-point sensor calibration procedure.

As shown in Fig. 6a, three clusters out of six consumed the total amount of oxygen in the chamber in less than 25 minutes. The corresponding pH curves indicate that the three fast respiring clusters show at least three kinetics with different slopes: 0 to 25 minutes (decreasing pH value), 25 to 50 minutes (nearly constant pH value), and 50 to 100 minutes (increasing pH value) (Fig. 6b). The corresponding MMP curves in Fig. 6c, represented as the intensity ratio of aggregates to monomers of the JC-1 dye I_red_/I_green_ (see Materials and Methods), indicate a depolarization (drop in the I_red_/I_green_ ratio) event of the mitochondrial membrane between 70 and 80 minutes. Cluster 4 (beige curve) completely consumed oxygen inside the microchamber at about 35 minutes; its pH variation in the first part of the kinetics (decreasing pH value) appears to end at about 25 minutes, after which it is mostly constant, with a slight increase starting at around 70–80 minutes and until the end of the measurement. The corresponding MMP value decreases towards the end of the measurement (beyond 60 minutes).

We observed differential dynamics of the MMP value, where some of the cell populations showed a marked depolarization of the mitochondrial membrane, while the others remained nearly constant over the entire duration of the assay (Fig. 6c). Interestingly, the observed decrease in MMP appears to be associated with OCR in that the slowly respiring cell populations (blue and red curves in Fig. 6a) maintained or even slightly increased their MMP level and the fast respiring populations (purple, pink and brown curves, OCR values of 0.635 fmoles·min^−1^·cell^−1^, 0.664 fmoles·min^−1^·cell^−1^, and 0.602 fmoles·min^−1^·cell^−1^, respectively) showed the most significant drop in MMP. The cell population with an intermediate OCR (beige curve) showed also an intermediate level of change in MMP. Furthermore, we observe delay of 20–30 minutes between the time point when all oxygen is consumed in the well and the marked drop in MMP occurs. Both observations may harbour functional relevance, but would need to be validated in other cell types in follow-up studies. Although purely speculative at this point, the observed dynamics of oxygen consumption and MMP might be explained by a molecular mechanism underlying mitochondrial function and oxygen availability. Low oxygen levels result in free radical generation such as reactive oxygen species and nitric oxide leading to oxidative stress of the cell. The oxidative stress has been shown to induce mitochondrial membrane permeabilization resulting in its depolarization^50^ which leads to apoptosis^51^.

**Figure 7:**
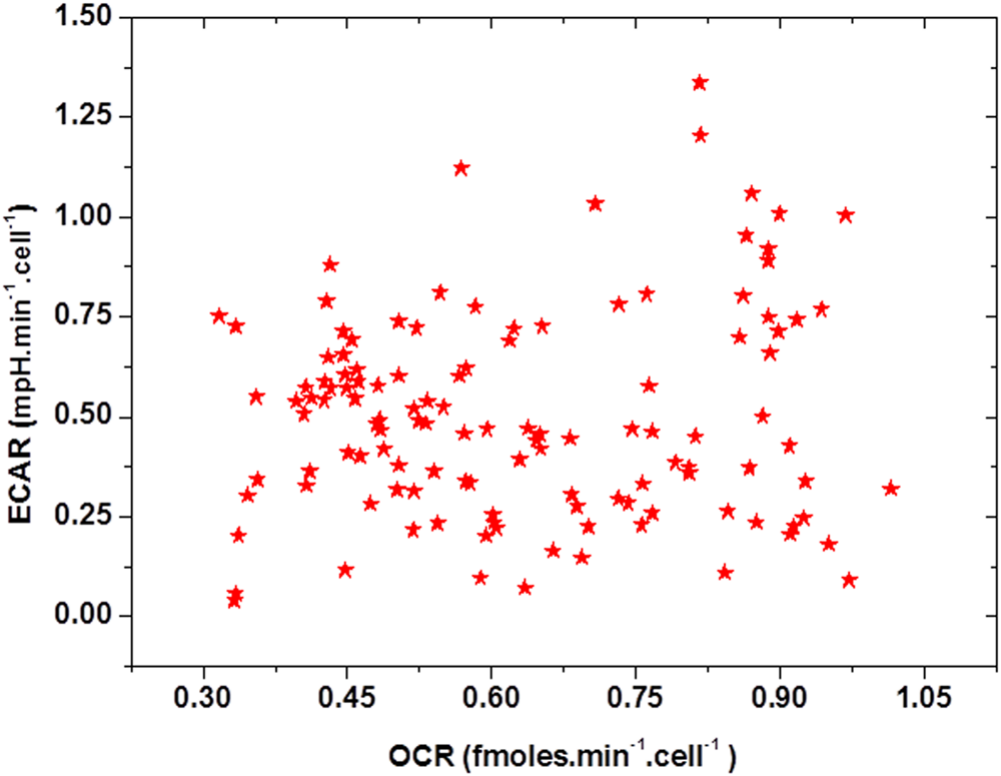
A relative cellular energy metabolism profile of the studied human esophageal epithelial (CP-D cell line) cells. For comparison purposes, the values of OCR and ECAR are reported normalized against the total number of cells per population. Each dot in the graph represents one cell population. Despite the signal averaging over the entire populations, marked heterogeneity among the different cell populations is noticeable, with some populations showing a predominantly oxidative phosphorylation-driven profile and some relying mostly on the glycolysis for bioenergy production. The majority of the populations exhibit varying degrees of OXPHOS and glycolysis.

Figure 7 shows the relationship between the OCR and ECAR. In spite of the averaging over clusters of cells, the OCR and ECAR relationship demonstrate a significant population-to-population heterogeneity. A detailed understanding of the interrelationship between OCR, ECAR, and MMP is beyond the scope of this work, and further studies are needed to enable deeper insight into the metabolic profile, and its alterations, in the context of pre-malignant progression.

In summary, we have demonstrated a new technology that enables real-time quantitative multiparameter functional profiling in small populations of cells, utilizing both extracellular and intracellular optical sensors. A scalable embedded micro-pocket array structure has been designed and fabricated on fused silica substrates to integrate multiple, spatially separated extracellular sensors for multiparameter analysis. Hermetically-sealed microchambers were created and demonstrated where the microchambers contain extracellular sensors that are deposited into the microchambers using a contact pin printer. The calibration and performance of the microsensors as well as the device functionality, in terms of real-time monitoring of extracellular and intracellular physiologic parameters, has been demonstrated. Further studies to fully understand the link between OCR, ECAR, and MMP, may help in developing new strategies for diagnostics and therapeutic development. The method presented here enables the development of specialized modules with similar architecture that, among other applications, can address: the co-culture phenotypic response of structured cell populations of different cell types, examination of fundamental cell signalling questions tied to chemotactic response to stimulus factors, and interrogation of inflammation mechanisms. Our future aims include further miniaturization and other adaptations of the device structure to facilitate simultaneous measurements, with single-cell resolution, of parameters including intracellular pH, reactive oxygen species (ROS) and other analytes such as K^+^, and Ca^+^ in addition to OCR, ECAR and MMP.

## Materials and Methods

### Cell culture

Experiments are performed with immortalized human esophageal epithelial cells (CP-D cell line) derived from patients with Barrett’s esophagus with dysplasia^52^. Cells were cultured in T75 tissue culture flasks (Corning, Corning, NY) to approximately 80% confluence, at which time they were trypsinized, centrifuged at 900 rpm for 3 minutes, and re-suspended in 2 mL of cell growth medium. The cells were grown at 37 °C, under 5% CO_2_ atmosphere, in cell culture flasks using GIBCO® Keratinocyte SFM cell growth medium (Invitrogen, Carlsbad, CA), supplemented with hEGF (Peprotech, Rocky Hill, NJ) at 2.5 µg/500 mL, BPE (bovine pituitary extract) at 25 mg/500 mL, and penicillin/streptomycin solution (Invitrogen) at 100 units/100 µg/mL. Cells were detached from the flask bottom using a 0.05% trypsin-EDTA solution and transferred to an Eppendorf tube for patterning. The working medium was obtained by adjusting the pH of DMEM (Dulbecco’s Modified Eagle Medium, Sigma Aldrich, St Louis, MO) to 7.4, using NaOH and HCl. The modified medium was supplemented with a penicillin/streptomycin solution, and stored in a cell culture incubator until use.

### Microchamber design

The microchamber consists of two parts: a bottom substrate with an array of equally spaced ECM spots, and a lipped lid with 9 micro-pockets for holding extracellular sensors. A side view of the chamber is shown in Fig. 1. Each micro-pocket is 60 µm in diameter and 15 µm deep, placed around the circumference of the lid, which has an inner diameter of 480 µm, with a lip width of 80 µm. Each such lid is aligned with a 3 × 3 array of wells (bottom substrate), with a center-to-center spacing of 700 µm (Fig. 2). The fused silica substrate is coated with a thin (3–4 µm) layer of Parylene-C, and patterned with fibronectin for selective cell adhesion. For experiments, each substrate is placed on a borosilicate disc that has been modified by gluing an acrylic ring around the circumference to mimic a Petri dish. This pseudo-dish is sterilized under UV light prior to assay. The lid is placed on a piston using an adhesive component such as polydimethylsiloxane (PDMS), after which the piston, with the lid, is aligned with the corresponding ECM patterns on the substrate under a microscope. A hermetic seal is produced by placing a 2.7 kg weight on top of the piston.

### Lid microfabrication

A schematic description of the major steps involved in the generation of a lid, along with dimensions and sensors, is shown in Fig. 2a. The microfabrication process involves an RCA cleaning step of 101.6 mm diameter double sided polished fused silica wafers (Mark Optics, Santa Ana, CA) to remove organic and inorganic residues. A 200 nm layer of silicon nitride was deposited onto the substrates as a masking layer using low pressure chemical vapor deposition (LPCVD: 250 mT, 835 °C, 100 sccm SiH_2_Cl_2_, 20 sccm NH_3_). A 1 µm layer of a positive photoresist (AZ 3312, Mays Chemical, Indianapolis, IN) was spin-coated and patterned onto the substrate, using standard photolithography techniques. This step defines the micro-pocket geometry inside the microwell lid. A reactive ion etch (RIE) was then performed to transfer the pattern into the Si_3_N_4_ layer by selective etching. The photoresist was removed using Microstripper 2001 (Columbus Chemical, Columbus, WI), and a 49% w/w hydrofluoric acid (HF) solution was used to wet etch the micro-pocket to a depth of 15 µm at room temperature. A second photolithography step was performed to define the lid microwell. A 3 µm layer of AZ 4330 (Mays Chemical, Indianapolis, IN) was used to achieve uniform coating and improve step coverage. A second RIE of Si_3_N_4_ was used to transfer the microwell pattern into the Si_3_N_4_ layer. The AZ 4330 was removed, and the 49% HF solution was used to etch the microwell to a depth of 30 µm. A third AZ4330 photolithography and Si_3_N_4_ dry etch, followed by photoresist strip, and HF wet etch, were performed to form the lip. The remaining Si_3_N_4_ was removed by the fourth RIE dry chemical etch, completing the microchamber lid fabrication. The wafer was diced into 3.5 × 3.5 mm pieces.

### Sensor deposition

The lids were cleaned in 70% isopropanol, sonicated in DI water for 30 minutes to remove particles from the lid surface, and then dried using nitrogen gas. The chips were then placed on a 1″ × 3″ microscope glass slide, and plasma cleaned for 60 min at 500 mTorr air atmosphere in a plasma oven (PlasmFlo PDC-FMG, Harrick Plasma, Ithaca, NY). Immediately after plasma cleaning, the chips were placed into a dry-seal glass vacuum desiccator (EW-06536–30, Cole-Parmer North America, Vernon Hills, IL) with trimethylsilylpropyl acrylate (475149, Sigma-Aldrich, St. Louis, MO) for vapor-phase silanization. The chips were silanized for three hours, under 28 in Hg pressure. The sensor material was then deposited into the lids, using a contact pin printing technology that was developed in-house. The deposition system consists of a print head with a free-floating micropipette (TIP 10TW1-L, World Precision Instruments, Sarasota, FL). Initially, the micropipette tip is dipped into a stock solution of the sensor, to allow the sensor to fill the tip of the micropipette by the capillary action. The tip is then positioned on top of the micro-pocket, and brought into contact with the silanized bottom of the micro-pocket, to allow a few pL of the sensor material to dispense into the micro-pocket^53^. Immediately after the deposition is complete, the sensor was cured at 80 °C for 18 hrs under a 100% nitrogen atmosphere. A fluorescent image of the lid, after deposition with oxygen and pH sensors, is shown in Fig. 2b.

### Cell patterning on the bottom substrate

The process flow of cell patterning is shown in Fig. 5a. Fused silica wafers were RCA cleaned to remove contaminants, and then coated with Parylene C using a Parylene deposition system (PDS 2010 Labcoater 2, Speciality Coating Systems, Indianapolis, Indiana). The deposition parameters were adjusted to produce a thin layer (3–4 µm) of Parylene C on the wafer, as per the manufacturer’s specifications. The coated wafer was then diced into 11 × 11 mm squares. These glass substrates were cleaned in a water sonication bath, followed by drying. The substrates were then plasma treated for 60 min, at 500 mTorr air pressure. The ECM protein fibronectin (F1141, Sigma-Aldrich St. Louis, MO) was diluted with water to yield 0.01% v/v concentration. Forty to fifty pL of the fibronectin solution were deposited per single location on the glass substrate in a pattern matching the lid array geometry. The deposition was performed by a non-contact piezoelectric dispensing robot (Rainmaker au301, Aurigin Technology Inc., Phoenix, AZ)^30^. Each location was deposited with two spots, and each spot was made up of two drops. The substrates with patterned fibronectin were then stored at 4 °C until further use.

For cell patterning, 50 µL of cell growth medium containing 10^5^ cells were dispensed onto the patterned substrates and incubated for 10 minutes at 37 °C, under 5% CO_2_ atmosphere. The substrates were then washed three times using PBS to remove the excess cells from the interstitial areas. Three washing steps were optimal to achieve 30–50 cells/spot with few interstitial cells, as shown in Fig. 5b. The average number of cells per cluster in our experiments was 40–50, and varied from as low as 30 to 80, with the upper limit set by the microwell size. The patterned substrates were incubated in the modified DMEM at 37 °C, under 5% CO_2_ atmosphere for 2–3 hours before starting the assay.

### Sensors

Oxygen consumption rates (OCRs) were monitored using a platinum porphyrin derivative, Pt (II) octaethylporphine (PtO534, Frontier Scientific, Logan, UT) as a phosphorescence sensor. The stock solution was prepared as reported previously^22^. pH was monitored using acryloylfluorescein as a fluorescent sensor material, which was synthesized as reported elsewhere^54^. Briefly, 180 mg of fluoresceinamine, and 60 µL of acryloylchloride were added to 20 mL of dry acetone, and stirred for 1 h in the dark. The precipitate was filtered and washed, first with acetone, and then with dichloromethane, to get 160 mg of the sensor monomer referred to as S2. The sensor stock solution was prepared by mixing 1 mg of S2, 800 mg of 2-hydroxyethyl methacrylate (477028, Sigma-Aldrich, St. Louis, MO), 150 mg of acrylamide (01696, Sigma-Aldrich), 50 mg of poly(ethylene glycol) dimethacrylate (409510, Sigma-Aldrich), 10 mg of azobisisobutyronitrile (755745, Sigma-Aldrich), and 250 mg of 4-arm poly(ethylene glycol) (A7020-1, JenKem Technology, Allen, TX) under stirring at room temperature, until a clear, homogenous and viscous solution was obtained. This solution was stored at 4 °C until further use.

Fluorescence labelling of the nuclear DNA and mitochondrial membrane potential in live cells was performed with Hoechst 33342 (H1399, Invitrogen, Grand Island, NY) and JC-1 (T-3168, Invitrogen) dyes, respectively. A working solution of 1 µg/mL of Hoechst 33342 was prepared per the manufacturer’s recommendations. The cells were stained and incubated for 30 minutes prior to experiment. A 1 mg/mL solution of JC-1 was prepared by mixing 2.5 mg of JC1 with 2.5 mL of DMSO. The cells were stained using a DMEM solution of 1 µg/mL of JC-1, made from the stock solution. The working solution of JC-1 was mixed thoroughly by vortexing for 60 minutes, using a vortex mixer (MixMate, Eppendorf, Hauppauge, NY). The cells were stained with JC-1 for 30 minutes, and washed three times with DMEM before the experiment. The amount of JC-1 inside the mitochondria increases with increasing hyperpolarization (more electrically negative interior) of the mitochondria and vice versa. The MMP sensing mechanism of JC-1 is based on changes in the spectral emission properties of the dye resulting from it forming the so-called J-aggregates that exhibit a sharp emission band in the red spectral region (590 nm) inside the mitochondria due to an increased dye concentration. As a result, one can utilize the ratio of fluorescence signal emitted by the J-aggregates and the monomers of the dye (green spectral region) to measure relative changes in MMP.

### Experimental setup

The physiological measurements were performed on an inverted microscope (TE2000, Nikon, Melville, NY). For hermetic seal creation the patterned substrates and the well arrays were first aligned and then sealed using a high precision XYZ translation stage and a motorized rotation stage mounted on the microscope stage. The extracellular pH and O_2_ sensors, and the intracellular JC-1 dye, were excited using a multi-LED illumination source (LED4C2, Thorlabs Inc, Newton, N.J) consisting of four LEDs emitting at central wavelengths of 405 nm, 470 nm, 530 nm, 617 nm, and coupled to the epi-illumination port of the microscope. Only 405 and 470 nm excitation wavelengths were used in the study. Excitation light in the wavelength range of 374–398 nm was used for O_2_ sensor excitation and was obtained by filtering the emission spectrum of the 405 nm LED with an excitation bandpass filter. The 470 nm LED was used to excite the pH sensor and JC-1 dye. A total of three images in three spectral channels corresponding to the emission maxima of the fluorophores were acquired. For excitation, two different excitation filters mounted in separate filter cubes in the microscope were used – a bandpass filter (FF01-386/23-25, Semrock Inc., Rochester, NY) for the O_2_ sensor, and a short pass filter (FF01-492/SP-25, Semrock) for excitation of the pH sensor and the JC-1 dye. Both filter cubes were equipped with identical dichroic beam splitters (FF506-Di03-25X36, Semrock Inc., Rochester, NY). The emission signal was passed through narrow bandpass emission filters (FF01-590/20-25 for JC-1 aggregates,FF01-640/14-25 for oxygen, and FF01-527/20-25 for pH and JC-1 monomers, all three from Semrock) mounted in a motorized filter wheel (Lambda 10–2, Sutter Instrument Company, Novato, CA), before being detected by an air-cooled electron-multiplying CCD (Cascade 512B, Photometrics, Tucson, AZ). Both LEDs were operated in a pulsed mode, and synchronized with the exposure time (20 ms) of the camera, to ensure that the sensors are only illuminated during the image collection cycles. The extracellular and intracellular sensors’ intensity data were extracted from the images by calculating the average pixel intensity in regions of interest (ROI) defined around the sensor areas, and cells, in the corresponding emission spectral channels. Each sensor image was acquired twice: once with the LED excitation, and the other without, for dynamic background correction. The background images were subtracted in real time from the images with the excitation turned on to correct for background contributions to the signal. Apart from this, an extra ROI was also placed in the interstitial area between the sensors, and its average intensity subtracted from the background-corrected images, to compensate for stray light reaching the camera. The data acquisition cycles, including the acquisition of the three sensor images (O_2_, pH and JC-1) and the three background images, were repeated every 8 seconds.

### Data analysis

Statistical analyses of the data, data fitting procedures, and ECAR were performed using OriginPro v. 8 (OriginLab Corp., Northampton, MA) software. Data analysis software for OCR determination was written using LabView 8.6 (National Instruments, Austin, TX).
